# Biomarker-Based Diagnosis and Risk Stratification in Sepsis-Associated Acute Kidney Injury: From Molecular Mechanisms to Multimarker Panels

**DOI:** 10.3390/diagnostics16091262

**Published:** 2026-04-23

**Authors:** Breallan De Jesús Romero Pajaro, Diana Carolina Caicedo Sánchez, Michael Mario Vélez Lora, John Freddy Mina Gasca, Damián Alberto Ochoa Guette, Geraldine Romero Martínez, Lileth Romero Pájaro, Álvaro José Viñas Granadillo, Juan Rodríguez-Macías

**Affiliations:** 1 Faculty of Health Sciences, Department of Internal Medicine, Universidad Libre Barranquilla Campus, Barranquilla 080001, Colombia; dianac-caicedos@unilibre.edu.co (D.C.C.S.); michaelm-velezl@unilibre.edu.co (M.M.V.L.); jhonf-minag@unilibre.edu.co (J.F.M.G.); damiana-ochoag@unilibre.edu.co (D.A.O.G.); aljovigran@hotmail.com (Á.J.V.G.); 2 Faculty of Medicine, Department of Anesthesiology, Universidad del Sinú Cartagena Campus, Cartagena 130001, Colombia; geraldineromeromartinez01@gmail.com; 3 Faculty of Medicine, Universidad de Cartagena, Cartagena 130015, Colombia; lromerop@unicartagena.edu.co; 4 Department of Nephrology and Transplantation, Clínica General del Norte, Barranquilla 080003, Colombia; 5 Facultad Ciencias de la Salud, Exactas y Naturales, Universidad Libre Barranquilla Campus, Barranquilla 080001, Colombia

**Keywords:** acute kidney injury, sepsis-associated acute kidney injury, biomarkers, risk stratification, multimarker panels, precision medicine

## Abstract

Sepsis-associated acute kidney injury (SA-AKI) remains a major diagnostic challenge in critically ill patients, as conventional functional criteria—serum creatinine and urine output—often detect AKI after clinically relevant pathophysiological derangement has already evolved. Increasing evidence suggests that SA-AKI reflects a heterogeneous process characterized by early cellular stress, microcirculatory dysfunction, inflammation-associated injury, and maladaptive repair preceding overt functional decline. In this context, biomarker-based approaches have been investigated to improve early risk stratification, phenotypic characterization, and prognostic assessment in septic patients. This narrative review synthesizes current evidence on established and emerging biomarkers relevant to SA-AKI, encompassing stress markers ([TIMP-2]•[IGFBP7]), tubular injury markers (e.g., NGAL, KIM-1, IL-18), functional markers (e.g., proenkephalin/penKid, cystatin C), and exploratory molecular signatures such as circulating microRNAs (miRNAs). We examine their temporal dynamics, performance estimates, and context-dependent applicability in sepsis, and discuss limitations related to heterogeneity, assay variability, and threshold standardization. Particular attention is given to multimodal and longitudinal strategies integrating biomarkers with KDIGO criteria and clinical phenotyping. Finally, we outline a stratified framework for biomarker interpretation in SA-AKI anchored to pathophysiological windows and clinical decision points. While available evidence supports the potential of selected biomarkers for short-term risk stratification and trajectory assessment, implementation requires prospective validation demonstrating incremental value beyond established models and measurable impact on patient-centered outcomes.

## 1. Introduction

Over recent decades, acute kidney injury (AKI) has emerged as one of the most frequent and severe complications affecting hospitalized patients, especially in critical care [1,2,3,4]. The prevalence of AKI has increased globally over the last 10 years, partly due to population aging, greater patient complexity, increased use of oncological therapies, and broader use of invasive procedures [5,6,7]. In intensive care units, AKI may occur in up to 50% of patients, with mortality rates exceeding 30% and reaching up to 60%, particularly in patients with sepsis [4,8,9].

In addition, AKI is associated with severe and potentially irreversible long-term consequences, including reduced functional capacity, increased rehospitalizations, higher cardiovascular risk, and a non-negligible risk of progression to chronic kidney disease [4,10,11,12]. Within the spectrum of AKI, sepsis represents one of the most frequent causes in the hospital setting, particularly in the intensive care unit [3,4]. This condition constitutes a specific and definable form of AKI, referred to as sepsis-associated AKI (SA-AKI), which is considered a distinct pathophysiological and clinical entity not solely attributable to systemic hypoperfusion or hypotension. Instead, SA-AKI is characterized by a predominant role of renal microcirculatory alterations, renal endothelial dysfunction, inflammation, oxidative stress, and mitochondrial dysfunction [3,4,6,13]. These processes can lead to heterogeneous tubular injury under conditions of apparently preserved renal blood flow, supporting the concept of a dissociation between global hemodynamic parameters and the development of AKI [4,6,11,14]. Therefore, SA-AKI should not be understood exclusively as the result of hemodynamic failure, but rather as the reflection of a complex cellular and molecular phenomenon determined by the host’s response to sepsis [4,8,10,14].

However, despite their clinical utility, the diagnosis of AKI in adults is currently based on traditional markers with well-recognized limitations. Table 1 compares classifications that use these markers [2,3,15]. Serum creatinine is a late marker influenced by muscle mass, hydration and volume status, medications, and analytical variability; elevations may occur 24 to 72 h after the onset of tubular injury [7,12,15]. Oliguria is also nonspecific and reflects functional alterations that, although potentially pronounced, do not necessarily correspond to structural renal damage [3,15,16].

Thus, the discordance between early AKI pathophysiology and its detection using late-response markers has driven the search for biomarkers capable of identifying subclinical kidney injury before overt functional loss. This concept of “subclinical AKI” has long attracted research interest and has gained relevance in critically ill patients, in whom the window for effective intervention is extremely short and progression to multiple organ failure may occur rapidly [3,4,7,12,15,16]. Multiple emerging biomarkers have been evaluated for early detection of AKI, including NGAL, KIM-1, IL-18, L-FABP, cystatin C, NAG, suPAR, and cell cycle arrest biomarkers [TIMP-2]•[IGFBP7] [3,4,10,15,16]. These biomarkers allow characterization of different aspects of kidney injury, such as structural damage, inflammation, cellular stress, apoptosis, and alterations in endothelial permeability [4,10,11,14,15,16].

NGAL and [TIMP-2]•[IGFBP7] have demonstrated value in clinical scenarios such as major surgery, sepsis, and postoperative cardiac care [4,6,16]. Consequently, they have been proposed as potential tools for risk stratification and preclinical detection of AKI. However, widespread implementation still faces significant barriers, including methodological variability, lack of standardized cutoff values, the influence of systemic inflammation, limited availability, and high cost [3,15,16]. Understanding the molecular pathways involved in AKI is essential to appropriately interpret the potential clinical use of these biomarkers [10,14]. This is particularly relevant in SA-AKI, where interactions among PAMPs, DAMPs, Toll-like receptors (TLRs), NF-κB activation, vascular dysfunction, and ultimately a mixed phenotype of functional and structural injury are observed [3,4,8,14,17].

The integrated analysis of biomarkers and molecular pathways also provides an opportunity to redefine the early diagnosis of AKI, improve outcome prediction, and inform individualized, risk-adapted supportive management in the adult septic patient [4,7,16]. Primary objective: to provide a pragmatic, time-sensitive framework for early risk stratification and trajectory-oriented assessment of adults with sepsis at risk of sepsis-associated acute kidney injury by integrating complementary biomarker domains. Secondary objectives: to map biomarker signals to key pathophysiological processes and sampling windows, and to synthesize analytical validity and implementation constraints that influence clinical interpretability and future research priorities.

To complement existing reviews, this review translates heterogeneous biomarker evidence in SA-AKI into an explicitly clinical-readiness-oriented synthesis. First, we integrate biomarker domains within an OCEBM-informed appraisal to distinguish clinical-grade tools from exploratory candidates and to avoid unsupported cross-study superiority claims. Second, we operationalize biomarker kinetics through a consolidated timing table to support sampling decisions in sepsis. Third, we propose a pragmatic longitudinal algorithm that links biomarker patterns to risk trajectories and monitoring intensity.

**Table 1:** AKI criteria according to major classifications and current validity.

System (Year)	Definition by Creatinine	Definition by Urine Output	Staging	Comment
RIFLE (2004)	Risk: SCr × 1.5 or ↓ GFR > 25%; Injury: SCr × 2 or ↓ GFR > 50%; Failure: SCr × 3 or ↓ GFR > 75% or SCr ≥ 4.0 mg/dL with ↑ ≥0.5 mg/dL	Risk: <0.5 mL/kg/h × 6 h; Injury: <0.5 mL/kg/h × 12 h; Failure: <0.3 mL/kg/h × 24 h or anuria × 12 h	3 severity classes (R/I/F) + 2 outcome classes (Loss; ESRD)	Useful for historical comparison and some studies; less used than KDIGO in current guidelines
AKIN (2007)	Stage 1: ↑ SCr ≥ 0.3 mg/dL or 1.5–2× baseline (within 48 h); Stage 2: 2–3×; Stage 3: ≥3× or SCr ≥ 4.0 mg/dL with acute ↑ ≥0.5 mg/dL or RRT	Stage 1: <0.5 mL/kg/h × 6 h; Stage 2: <0.5 mL/kg/h × 12 h; Stage 3: <0.3 mL/kg/h × 24 h or anuria × 12 h	3 stages (1–3)	Transitional; many older cohorts report it, but currently KDIGO is preferred
KDIGO AKI (2012)	AKI if: ↑ SCr ≥ 0.3 mg/dL within 48 h or ↑ SCr ≥ 1.5× baseline (presumed within ≤7 days) or initiation of RRT	<0.5 mL/kg/h for ≥6 h	Stage 1: 1.5–1.9× or ≥0.3 mg/dL; UO < 0.5 × 6–12 h; Stage 2: 2.0–2.9×; UO < 0.5 ×≥12 h; Stage 3: ≥3× or SCr ≥ 4.0 mg/dL or RRT; UO < 0.3×≥24 h or anuria ≥12 h	Dominant framework in clinical practice, research, and trials

Adapted from [3,4,8,18,19,20,21]. In 2025, KDIGO 2012 remains the operational standard for AKI definition and staging, while consensus and nomenclature documents have reinforced the AKI–AKD–CKD continuum and terminological standardization, without replacing the classic KDIGO thresholds. Abbreviations: AKI = acute kidney injury; RIFLE = Risk, Injury, Failure, Loss, End-stage kidney disease; AKIN = Acute Kidney Injury Network; KDIGO = Kidney Disease Improving Global Outcomes; SCr = serum creatinine; GFR = glomerular filtration rate; RRT = renal replacement therapy; UO = urine output; Loss = persistent loss of kidney function for >4 weeks; ESRD = end-stage renal disease, persistent loss of kidney function for >3 months; ↑ = increase; ↓ = decrease.

## 2. Materials and Methods

### 2.1. Review Design

This manuscript is a state-of-the-art narrative review with a structured literature search. It was designed to synthesize conceptual, pathophysiological, and clinically oriented evidence on early acute kidney injury (AKI) biomarkers and the principal molecular pathways involved, with a particular focus on sepsis-associated AKI in adult critical care. A narrative approach was selected because the topic spans heterogeneous evidence streams (translational and experimental research, observational cohorts, clinical trials, prior reviews, and consensus/guideline documents), where a quantitative pooling strategy is frequently inappropriate due to differences in biomarker platforms, sampling windows, AKI definitions, and clinical contexts.

To strengthen methodological quality within a narrative framework, the manuscript was developed in alignment with the SANRA (Scale for the Assessment of Narrative Review Articles) domains as a qualitative guiding framework (i.e., no numeric SANRA scoring was performed), emphasizing a clearly justified scope, comprehensive and relevant literature coverage, balanced critical appraisal, and coherent argumentation.

### 2.2. Information Sources and Search Strategy

A structured search was conducted in PubMed/MEDLINE, Scopus, Web of Science, and Ovid. Google Scholar was used as a complementary source for citation chasing (backward and forward searches) to identify influential or highly cited items not captured by indexed databases. Reference lists from key articles and consensus documents were also manually screened.

The search period covered the years 2004 to 2025, spanning the introduction of the RIFLE classification and the emergence of early AKI biomarkers through the most recent evidence available at the time of manuscript preparation.

MeSH terms and free-text keywords were combined and adapted to each database using Boolean operators (AND/OR). Core search concepts included acute kidney injury; sepsis-associated AKI; biomarkers/early biomarkers; kidney stress; NGAL; KIM-1; TIMP-2; IGFBP7; cell cycle arrest; IL-18; suPAR; L-FABP; cystatin C; endothelial dysfunction; inflammation; oxidative stress; mitochondrial dysfunction; and regulated cell death.

The evidence was organized a priori into four thematic axes:

(i). Early AKI biomarkers grouped by biological domain (structural injury, tubular stress, inflammation/endothelial dysfunction, and functional change);
(ii). Molecular pathways underpinning SA-AKI;
(iii). Sepsis-specific immunological and microcirculatory mechanisms shaping biomarker kinetics and interpretation;
(iv). Clinical integration considerations (timing, feasibility, and decision-support implications).

### 2.3. Scope Boundaries and Eligibility Considerations

The review prioritized the adult (≥18 years) literature relevant to early AKI detection and risk stratification, including biomarkers reflecting structural injury, tubular stress, inflammation/endothelial activation, or early functional change that may precede or complement serum creatinine and urine output. Mechanistic studies were included when they provided biologically plausible links to biomarker release/kinetics and were translatable to adult AKI/SA-AKI. Evidence directly addressing SA-AKI was prioritized; when SA-AKI-specific evidence was limited, broader critical-care AKI research was used with explicit labeling as extrapolated.

Non-informative formats without original scientific content (e.g., opinion-only commentaries, conference abstracts without sufficient data) were not prioritized. Pediatric-only studies were excluded. Animal studies were selectively considered only when they provided foundational mechanistic insights directly informing biomarker biology or AKI pathways with clear relevance to adult disease.

### 2.4. Evidence Synthesis and Clinical-Readiness Framing

Evidence synthesis was qualitative and integrative. Findings were extracted and narratively summarized around (a) biomarker biology and matrix (urine/plasma); (b) clinically relevant sampling windows and kinetics; (c) diagnostic/prognostic performance as reported in critical-care cohorts; (d) sepsis-specific interpretative caveats; and (e) implementation barriers (assay variability, confounding, feasibility, and cost).

To facilitate clinical interpretability and to explicitly distinguish validated tools from emerging candidates, biomarkers and approaches were discussed using a pragmatic “clinical readiness” lens: (Tier 1) biomarkers with consistent performance across multiple adult critical-care cohorts and/or external validation with available assays; (Tier 2) biomarkers with promising but less consistent or context-dependent clinical evidence; and (Tier 3) exploratory candidates (including multi-omics signals) requiring further validation before clinical deployment.

## 3. Early AKI Biomarkers

In general terms, biomarkers can be grouped into four main categories: (1) tubular injury markers, (2) cellular stress markers, (3) renal function markers, and (4) inflammatory markers. This classification encompasses the predominant mechanisms described. Although none of these biomarkers is ideal for all clinical scenarios, their combined use may provide faster and more accurate diagnosis [22]. Below, the most extensively studied novel early AKI biomarkers in adults and their clinical applications are described.

### 3.1. General Classification

Table 2 and Table 3 summarize key biomarkers used for early AKI detection.

### 3.2. Neutrophil Gelatinase-Associated Lipocalin (NGAL)

NGAL is a protein released by neutrophils and by injured tubular epithelial cells [16]. In the setting of ischemic kidney injury, NGAL levels increase rapidly in both plasma and urine, rising within a few hours as an early signal of tubular stress and inflammation [22,27].

In critically ill patients with sepsis, reported sensitivity ranges from 75% to 89% and specificity ranges from 70% to 85%. NGAL is widely used as an early AKI biomarker [22]. It has also been used to distinguish between transient and persistent AKI and has proven useful for the early identification of AKI secondary to cisplatin and amphotericin B, occurring 4.5 and 3 days earlier, respectively, than increases in serum creatinine [28,29,30].

### 3.3. Kidney Injury Molecule-1 (KIM-1)

KIM-1 is a transmembrane glycoprotein produced by proximal tubule cells and released into the urine after tubular injury; its concentration increases approximately 12 to 24 h after injury [23,31]. It can be used not only to determine the presence of AKI but also its severity and recovery potential, enabling earlier recognition, supportive measures, and closer monitoring [23]. It has been shown to be a biomarker of drug-induced AKI, like NGAL [29,32], including vancomycin-associated AKI, in which both are significantly elevated [33].

### 3.4. Tissue Inhibitor of Metalloproteinases-2 (TIMP-2) • Insulin-like Growth Factor–Binding Protein 7 (IGFBP7)

These two biomarkers can detect cell-cycle arrest in tubular epithelial cells and are released into the urine in the presence of renal stress [16,34]. Their levels increase 24 to 48 h before AKI can be identified by traditional functional markers [16,35,36,37], and they allow anticipation of a higher risk of requiring renal replacement therapy, as well as prolonged dependence on it [16,38].

### 3.5. Other Markers

*Cystatin C* is a functional biomarker that acts as a cysteine protease inhibitor and is released by nucleated cells. It is not affected by loss of muscle mass and therefore can provide a more accurate estimate of glomerular filtration rate after critical illness [3,39].

*Urinary C-C motif chemokine ligand 14 (CCL14)* is a biomarker of renal inflammation that indicates the persistence of severe AKI [3]. Elevated levels predict persistent stage 3 AKI and have been associated with a lack of renal recovery within the RUBY cohort [40]. In addition, CCL14 has been associated with progression to end-stage kidney disease in patients with diabetes mellitus [41].

*Interleukin-18 (IL-18)* is a proinflammatory cytokine that stimulates interferon-gamma release and can be detected in urine after acute proximal tubular injury [23,42].

*Urinary liver-type fatty acid–binding protein (L-FABP)* is a cytoplasmic protein expressed in proximal tubular epithelial cells. Its expression increases in response to hypoxia, oxidative stress, and ischemia [43]. It has been validated as a biomarker of AKI, particularly in perioperative states, contrast-induced nephropathy, and critically ill patients [44].

Processes of epithelial stress, lysosomal injury, apoptosis, and necroptosis of the proximal tubule induce urinary release of enzymes and structural proteins that function as early detectors of tubular injury; among these, *N-acetyl-β-D-glucosaminidase (NAG)* is noteworthy. NAG is a lysosomal hydrolase whose increased urinary excretion correlates with acute tubular injury before alterations become evident in traditional filtration parameters [3,8,13]. Its release occurs in parallel with oxidative stress, mitochondrial disruption, and regulated cell death [15,16,23].

Glomerular biomarkers reflect alterations in glomerular filtration. One biomarker in this group is *proenkephalin A 119–159 (penKid)*, which has been studied in critically ill patients. Dépret et al. showed that penKid identifies subclinical AKI, predicting mortality, progression to established AKI, and the need for vasopressor support independently of estimated glomerular filtration rate [26].

*Soluble urokinase-type plasminogen activator receptor (suPAR)* reflects systemic immune activation and inflammatory burden. Transcriptomic studies of sepsis-associated AKI demonstrate upregulation of inflammatory pathways involving monocyte and macrophage activation, supporting its role as a marker of immune dysregulation. Consequently, suPAR may be conceptually positioned as a systemic biomarker associated with inflammatory burden and risk of disease progression in multiorgan injury [3,8,13,17,45]. Another immune-related biomarker is urinary sTREM-1, which reflects neutrophil activation and highlights the importance of myeloid pathways in infection-associated AKI [8,13,17,46].

### 3.6. Multimarker Panels and Omics-Based Approaches for Early AKI Detection

The development of molecular, transcriptomic, and proteomic techniques has enabled a more precise characterization of the fundamental mechanisms underlying acute kidney injury (AKI), fostering the emergence of multimarker panels and omics-based approaches that overcome the diagnostic limitations of individual biomarkers [11,24,46]. The recent literature emphasizes that AKI pathophysiology is inherently heterogeneous; no single biomarker captures this complexity, thereby promoting the development of combinatorial strategies based on multicomponent molecular signatures or panels [11,24,47].

#### 3.6.1. Protein Biomarker-Based Panels

Among emerging multimarker strategies, one of the most extensively studied approaches is the combination of cellular stress biomarkers, such as [TIMP-2]•[IGFBP7] [15,16]. Additionally, some studies extend this rationale by integrating panels that combine NGAL, KIM-1, and L-FABP, aiming to simultaneously capture tubular injury, inflammatory activity, and alterations in renal function [3,25].

#### 3.6.2. Panels Based on Transcriptomic and Cellular Approaches

The introduction of transcriptomics has enabled the identification of subsets of renal epithelial cells with distinctive transcriptional profiles during AKI [7,14]. Hinze and Schmidt-Ott describe how techniques such as single-cell RNA sequencing (scRNA-seq) allow the detection of intermediate epithelial injury states, stressed-but-viable cells, early activation of repair-related genes, and the emergence of inflammatory subpopulations [24]. These findings enable the derivation of genetic signatures that facilitate early identification of trajectories, leading either to recovery or to progression toward chronic kidney disease (CKD).

#### 3.6.3. Integrated Omics Approaches

Proteomics, metabolomics, and epigenomics (including lactylation and other post-translational modifications) provide complementary information on mechanisms of injury in sepsis and other causes of AKI [3,13,48]. Lactylation of renal epithelial proteins correlates with the severity of inflammation and mitochondrial dysfunction [4].

#### 3.6.4. MicroRNAs as Components of Multimarker Panels

Bakinowska et al. provide evidence supporting the role of key regulatory miRNAs (miR-21, miR-155, miR-210, among others) in the pathophysiology of acute kidney injury. These small non-coding RNAs act as post-transcriptional regulators and participate in inflammation, hypoxic stress responses, and apoptosis [10].

Given their early detectability in serum and urine, miRNAs are considered promising candidates for incorporation into multimarker panels combining proteomic, transcriptomic, and metabolomic signatures [10,15,25]. Their structural stability, resistance to degradation, and relative tissue specificity further support their inclusion as valuable components of emerging molecular diagnostic strategies [10,23].

### 3.7. Clinical Utility: Early Detection, Risk Stratification, and Recognition of Subclinical AKI

Studies in critically ill patients have shown that early elevations of biomarkers are associated with an increased risk of progression to moderate-to-severe AKI, greater need for hemodynamic support, and higher mortality [3,16].

They may also reflect specific inflammatory or mitochondrial signatures, facilitating phenotypic stratification [3,8,13,17,48].

#### Utility in Prognostic Models and Precision Medicine

The integration of biomarkers with clinical variables improves risk discrimination [3,4,16]. For example, models combining cellular stress markers, inflammatory biomarkers, and transcriptomic panels enable differentiation between adaptive repair and progression to fibrosis, a key determinant of the transition to chronic kidney disease [10,11,14,49].

These findings indicate that the main value of biomarkers lies not in their isolated use but in integrated strategies embedded within dynamic risk algorithms that can be incorporated into clinical decision-support systems.

### 3.8. When Should Biomarkers Be Measured?

Available evidence supports a time-dependent strategy guided by clinical risk and the suspected timing of kidney injury:

- At hospital admission or when sepsis is suspected:

Cycle arrest biomarkers may be detectable within the first 12 h after injury and have been validated using cutoff values for early risk stratification [38,43].

- Within the first 6–12 h:

Markers of tubular damage such as NGAL rise rapidly (often within 2–6 h) and may increase in plasma and urine up to 24–48 h earlier than conventional functional criteria [50,51].

- Between 12 and 24 h:

Cystatin C typically increases within 12–24 h after AKI onset and has a short half-life (≈1.5 h), supporting early detection and dynamic monitoring [50,52,53].

- After resuscitation or hemodynamic stabilization:

Repeat biomarker assessment following hemodynamic optimization may differentiate transient functional alterations from persistent structural damage, with important prognostic and follow-up implications [23].

#### 3.8.1. What Is the Recommended Minimum Biomarker Panel?

Because AKI involves cellular stress, structural tubular injury, and functional decline, reliance on a single biomarker is often insufficient. A pragmatic integrated panel may therefore include the following:

*Renal stress biomarker*:

Urinary [TIMP-2]•[IGFBP7], expressed as (ng/mL)^2^/1000, has been widely used for early risk stratification of moderate-to-severe AKI in critically ill patients, with validated cutoffs of ≤0.3 (low risk), >0.3–≤2.0 (moderate risk), and >2.0 (high risk) [38,43].

*Tubular injury biomarker*:

NGAL demonstrates good discriminative performance with cutoff values associated with adverse renal outcomes [54], whereas KIM-1 typically rises within 12–24 h and peaks at 24–48 h after injury [23,50].

*Functional biomarker*:

Cystatin C has been associated with AKI development and impaired renal recovery, with persistently elevated levels observed in non-recovering patients [53].

This multimodal approach is consistent with the ADQI 23 conceptual framework and emphasizes the integration of biomarkers across complementary pathophysiological domains [23].

#### 3.8.2. How Should Biomarkers Be Interpreted? Limitations and Confounding Factors

Clinical interpretation of biomarkers should consider relevant limitations and potential confounders.

NGAL, KIM-1, and IL-18 may be elevated in states of systemic inflammation, infection, and multiorgan dysfunction independently of kidney injury, reducing specificity [23,50,55]. IL-18 has limited organ specificity, which constrains its use as a stand-alone marker; however, its predictive performance improves when combined with other tubular injury biomarkers [43,56].

Patients with chronic kidney disease may exhibit elevated baseline levels of NGAL, KIM-1, and L-FABP. Therefore, dynamic changes and temporal trends are often more informative than absolute values in isolation [23,43].

Age, sex, body composition, hypoalbuminemia, systemic inflammation, and high-dose corticosteroid therapy may influence serum cystatin C levels [23,53]. In severe sepsis, cystatin C may increase independently of renal function, potentially limiting its predictive value when used in isolation [57,58].

## 4. Pathophysiological and Molecular Pathways of Acute Kidney Injury

Acute kidney injury is a clinical syndrome characterized by convergent mechanisms that include tubular epithelial injury, endothelial dysfunction, and microcirculatory derangements, with important contributions from oxidative stress, mitochondrial dysfunction, and regulated cell death [3,4,7,21,59].

### 4.1. Ischemia–Reperfusion, Nephrotoxins, Endothelial Injury, and Microcirculatory Dysfunction

In ischemic AKI, reduced renal blood flow together with decreased perfusion pressure leads to medullary hypoxia, ATP depletion, and failure of ionic pumps, resulting in loss of epithelial polarity, cellular edema, and detachment of tubular cells into the lumen [3,46,60]. The reperfusion phase is commonly associated with a burst of reactive oxygen species (ROS), activation of endothelial cells and leukocytes, release of mitochondrial damage-associated molecular patterns (DAMPs), and amplification of inflammatory responses [3,59]. Mitochondrial dysfunction represents a central mechanistic axis in experimental models [7,59].

Nephrotoxins contribute additional mechanisms of direct cellular injury. In drug-associated AKI due to agents such as vancomycin, three predominant histological patterns have been described: acute tubular necrosis, acute tubulointerstitial nephritis, and crystalline cast obstruction. These patterns are thought to be mediated by intracellular drug accumulation in proximal tubular cells, which may induce oxidative stress, mitochondrial dysfunction, complement activation, and apoptosis [3,49,61]. Likewise, non-steroidal anti-inflammatory drugs are associated with afferent arteriolar vasoconstriction and reduced medullary blood flow due to prostaglandin inhibition, with possible additional tubular epithelial effects in selected contexts [3,62]. Iodinated contrast media exert combined effects that include intrarenal vasoconstriction, medullary hypoxia, free radical formation, and direct tubular toxicity, particularly in the presence of sepsis, hypovolemia, or pre-existing renal comorbidities [5,49,61,63].

Endothelial injury and microcirculatory dysfunction are widely recognized as key components of AKI across clinical settings [3,6,13,64,65,66].

### 4.2. Transition from Acute Kidney Injury to Chronic Kidney Disease

Progression from AKI to chronic kidney disease is increasingly recognized as an active biological process rather than a passive consequence of injury. Failed tubular epithelial repair, persistence of subclinical inflammation, and microvascular dysfunction appear to be major contributors to this transition [3,4,18]. AKI may therefore be conceptualized as part of a continuum that includes phases of injury, adaptive repair, maladaptive repair, and ultimately chronicity, characterized by activation of interstitial fibroblasts, extracellular matrix expansion, and progressive fibrotic remodeling [7,8,11].

**Figure 1:**
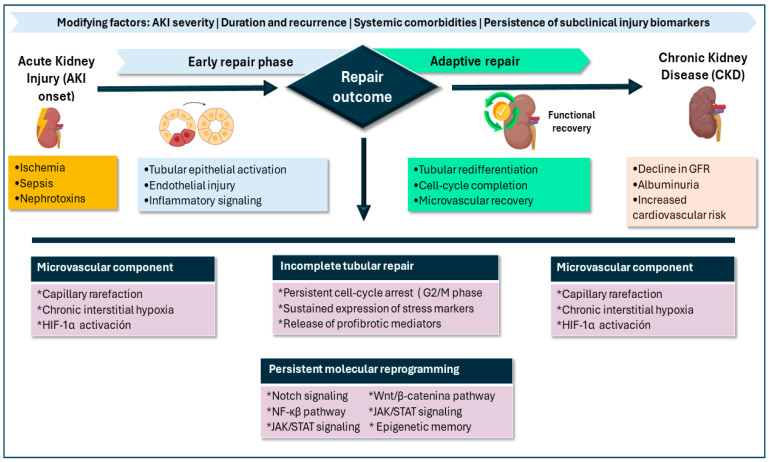
This schematic illustrates the dynamic and biologically active transition from acute kidney injury (AKI) to chronic kidney disease (CKD). Following an acute renal insult, an early repair phase characterized by tubular epithelial activation, endothelial stress, and inflammatory responses is initiated. The subsequent repair outcome represents a critical bifurcation point, leading either to adaptive repair—with tubular redifferentiation, cell-cycle completion, microvascular recovery, and functional restoration—or to maladaptive repair. Maladaptive repair is driven by incomplete tubular epithelial recovery, persistent cell-cycle arrest (G2/M phase), sustained expression of stress markers, and release of profibrotic mediators, in parallel with microvascular dysfunction, capillary rarefaction, and chronic interstitial hypoxia mediated by HIF-1α activation. Abbreviations: AKI, acute kidney injury; ARF, acute renal failure; CKD, chronic kidney disease; G2/M, G2/M phase of the cell cycle; HIF-1α, hypoxia-inducible factor 1 alpha; NF-κB, nuclear factor kappa B; JAK/STAT, Janus kinase/signal transducer and activator of transcription; Wnt, Wingless-related integration site signaling pathway; β-catenin, beta-catenin. Adapted from [7,11,24]. (All free elements in the figure originated from Servier Medical Art, http://smart.servier.com. Servier Medical Art by Servier is licensed under a Creative Commons Attribution 4.0 (https://creativecommons.org/licenses/by/4.0, accessed on 5 January 2026).)

Loss of microvascular integrity has been associated with injury irreversibility and correlates with progressive decline in glomerular filtration in both experimental models and patients with severe AKI. At the molecular level, the AKI-to-CKD transition involves persistent epigenetic and transcriptomic reprogramming, which may translate into sustained structural and functional vulnerability even after apparent clinical recovery [59]. Within this framework, maladaptive programs may involve prolonged activation of developmental and inflammatory signaling pathways—including Notch/Wnt, NF-κB, and JAK/STAT—as summarized in Figure 1 [7,11,24].

## 5. Sepsis-Associated Acute Kidney Injury

Current concepts of SA-AKI have evolved with recognition of the pathophysiological contribution of immunoinflammatory, microvascular, cellular, and metabolic components. Unlike other forms of AKI, SA-AKI is characterized by predominantly functional, potentially reversible tubular epithelial dysfunction, which may be present even in the absence of global renal hypoperfusion or massive cell death [4,8,67,68]. A major pathophysiological driver is activation of the innate immune system, linked to signaling through receptors such as Toll-like receptors and NOD-like receptors (TLRs and NLRs, respectively), which are stimulated by PAMPs or DAMPs.

This initiates intracellular signaling pathways mediated by NF-κB, IRF3, and other cascades, with a marked release of proinflammatory cytokines such as IL-6, TNF-α, and IL-1β. Systemically, this promotes inflammation, and within the kidney it translates into leukocyte diapedesis, endothelial dysfunction, increased vascular permeability, and microthrombus formation, impairing microcirculatory flow distribution and perpetuating tubular dysfunction [4,67,69]. A key factor in SA-AKI pathogenesis is renal microcirculatory dysfunction, which can lead to tissue hypoxia, heterogeneous distribution of peritubular capillary flow, and impaired glomerular autoregulation [4,8,67,68,69].

These alterations can generate focal regions of hypoxia/ischemia that, together with inflammation, disrupt tubular homeostasis. Consequently, proximal tubular epithelial cells respond to septic injury with phenotypic and metabolic reprogramming characterized by loss of polarity, cytoskeletal reorganization, and a shift toward aerobic glycolysis—initially adaptive and aimed at preserving cell viability under stress. However, if prolonged, this response may favor activation of proapoptotic pathways, fibrosis, and progression toward chronic dysfunction [68,69].

### 5.1. Immunological Interaction

In SA-AKI, the immune response is configured as a highly regulated process driven by exogenous and endogenous molecular patterns. TLRs—particularly TLR4 and TLR2—recognize microbial components such as lipopolysaccharide (LPS) and bacterial lipoproteins, respectively. This triggers intracellular pathways such as MyD88 and TRIF (Myeloid Differentiation Primary Response 88 and TIR-domain-containing adapter-inducing interferon-beta), which converge on activation of transcription factors including NF-κB and IRF3. This promotes transcription of proinflammatory mediators (e.g., TNF-α, IL-6, CCL2, and CXCL1), sustaining a chemotactic and proinflammatory milieu within the “septic kidney” [13,17,46,48]. In 2022, Guo et al., using transcriptomic analyses in murine models, documented that TLR2 and TLR4 are overexpressed in septic renal tissue together with their intracellular adapters, correlating with a significant increase in NF-κB-dependent gene transcription [46].

Beyond PAMPs, DAMPs play a central role in perpetuating inflammatory activation. Release of HMGB1, extracellular ATP, free histones, and mitochondrial DNA from injured renal cells can reactivate TLR2, TLR4, and TLR9, generating a feedback loop that amplifies proinflammatory signaling even in the absence of ongoing infectious stimuli [13,17,48]. This also promotes activation of the NLRP3 inflammasome, whose stimulation by ROS and DAMPs drives IL-1β maturation and caspase-1 activation, thereby contributing to progressive tubular injury [17,46].

TLR-mediated signaling operates in coordination with other cellular pathways such as MAPK (p38, JNK) and JAK–STAT, which amplify inflammatory responses and shape local immune polarization. This promotes expression of ICAM-1 and VCAM-1, facilitating leukocyte recruitment and activation in the renal cortex [13,46]. Proximal tubular cells function not only as targets of injury but also as active immune effectors; by expressing multiple TLRs, they respond by secreting CXCL1, IL-8, and CCL2, and by increasing ROS generation, particularly after synergistic exposure to LPS and HMGB1 [13,17,48].

Histone lactylation has been identified as a bridge between sepsis-induced metabolic reprogramming and immune activation. In 2025, Jiang et al. described how intracellular lactate generated during sepsis induces H3K18 lactylation, thereby enhancing expression of NF-κB-regulated inflammatory genes such as TNF-α and IL-6 [48]. In this process, infiltrating macrophages, under stimulation of the TLR–NF-κB axis, may adopt a sustained M1 phenotype, hindering resolution of injury [17,48].

### 5.2. Biomarker Dynamics

A pathophysiology-based framework helps align biomarker behavior with the dominant injury axes in SA-AKI. Ischemic or toxic tubular epithelial injury is reflected by elevations in tubular injury biomarkers, which may help distinguish structural tubular damage from predominantly functional alterations in clinical settings such as cirrhosis, sepsis, and surgery [3,16,23,65]. However, in sepsis, these biomarkers may show reduced specificity, as systemic inflammation can increase their levels even in the absence of primary renal involvement; therefore, results should be interpreted within the broader clinical context [4,68,70].

In parallel, the stress biomarker [TIMP-2]•[IGFBP7]—highlighted by the ADQI group for its predictive value in critically ill patients—may support recognition of a subclinical phase of injury and provide a potential window for targeted preventive interventions [70].

The inflammatory signature described in SA-AKI is conceptually reflected by biomarkers such as IL-18 and selected miRNAs, as well as systemic mediators including suPAR and sTREM-1 [4,10,13,17,46].

Other biomarkers may complement risk stratification in septic patients. For example, penKid and cystatin C may represent an alternative for estimating glomerular filtration in sepsis [70].

Beyond kidney-specific biomarkers, routine biochemical parameters have also been explored as indirect indicators of renal risk. Hyperchloremia has been associated with a higher incidence of AKI; in this regard, León-Miranda et al. documented a correlation between elevated chloride levels and impaired AKI recovery, suggesting possible links to renal vasoconstriction and interstitial edema [71].

### 5.3. Clinical Implications: Stratification and Decision Making

Sepsis-associated AKI can be framed as a real-time decision-making challenge in which trajectory and context often matter at least as much as static thresholds. ADQI 28 emphasizes that the pathophysiological heterogeneity of SA-AKI requires moving beyond a classic, static approach toward a more dynamic and integrative assessment [4].

Within this paradigm shift, Legrand et al. describe an operational precision-medicine framework that structures clinical language and links it directly to clinical decision making: “phenotype” as what is observable, “endotype” as the underlying biological mechanism, “subphenotype” as clinical segmentation within the phenotype, and “treatable trait” as the anchoring point between stratification and targeted therapies [72].

This taxonomy is not merely conceptual; it supports the distinction between variables that predict risk (prognostic indicators) and those that may predict treatment response (predictive indicators), helping explain how a heterogeneity of treatment effect can dilute the benefit when an “average” intervention is applied to a biologically diverse population [72].

Consistent with this, ADQI 28 proposes functional stratification across three levels—subclinical AKI, reversible functional injury, and established damage—aimed at identifying preventive windows before injury becomes “visible” according to conventional criteria [4]. In gray-zone scenarios, integrating biomarkers with physiological assessment may help reduce both overtreatment and therapeutic inertia [4,70].

In daily practice, these principles translate into concrete decisions, particularly in fluid management. Nordin et al. propose treating fluids as a “drug” (type, dose, duration, and de-escalation), emphasizing the need to prevent fluid resuscitation from progressing into sustained venous congestion—a mechanism that can worsen effective renal perfusion and hinder recovery [73].

A particularly sensitive—and debated—decision is the initiation of RRT. The IDEAL-ICU trial is clinically informative because it compared early initiation with a delayed strategy incorporating rescue criteria in septic patients with severe AKI without an initial urgent indication. Its findings suggest that a substantial proportion of patients can avoid RRT under close monitoring, supporting an approach that emphasizes clinical trajectory, in which biomarkers may contribute to ongoing risk reassessment [74].

Overall, trajectory- and subphenotype-based stratification, supported by biomarker domains summarized in Table 4, may provide a pragmatic bridge between biological complexity and more proportional, anticipatory clinical decision making centered on renal recovery [72].

## 6. Therapeutic Management of Sepsis-Associated Acute Kidney Injury

Although this is not a primary aim of the present review, it is important to emphasize that the management of sepsis-associated acute kidney injury remains anchored in supportive care, prevention of progression, and timely treatment of the underlying cause. Despite advances in mechanistic understanding, there is currently no universally effective pharmacologic therapy for SA-AKI; therefore, management is typically individualized according to clinical context, dominant pathophysiological axes, and injury severity [3,4,77,78,79].

### 6.1. Supportive Care and Nephroprotective Measures

Initial management is integrated within sepsis care (source control, appropriate antimicrobials, and organ support), while simultaneously minimizing kidney “second hits.” Contemporary frameworks emphasize pragmatic implementation of nephroprotective measures—particularly in high-risk septic patients—including minimizing exposure to nephrotoxins when feasible, avoidance of sustained hyperglycemia, consideration of alternatives to radiocontrast when appropriate, and hemodynamic monitoring with optimization of both volume status and perfusion [4,64,79].

### 6.2. Hemodynamic and Fluid Strategy

Because SA-AKI may occur despite apparently preserved macrohemodynamics, supportive care should be individualized and repeatedly reassessed, integrating perfusion goals with prevention of iatrogenic fluid overload and venous congestion [4,73].

In this context, balanced crystalloids are generally preferred over chloride-rich solutions, and synthetic colloids should be avoided in septic patients at risk for AKI; albumin-containing strategies may be considered only in selected scenarios after large crystalloid volumes, recognizing that high-quality evidence for kidney benefit remains limited [79]. While optimal hemodynamic targets remain debated, a mean arterial pressure (MAP) ≥65 mmHg is commonly used as a pragmatic minimum to support organ perfusion, with higher targets potentially relevant in selected patients (e.g., chronic hypertension) [79].

### 6.3. Biomarker-Informed Risk Windows to Support Clinical Decision Making

Biomarkers are not therapies; however, they can support the timing and proportionality of management decisions by identifying risk windows that precede conventional criteria. An integrative approach combines functional markers with injury/stress biomarkers to refine detection of subclinical or evolving SA-AKI and to inform the intensity of monitoring and preventive strategies [4,70,79]. This approach is conceptually aligned with functional stratification that prioritizes trajectory over static thresholds [4].

### 6.4. Renal Replacement Therapy: Indications, Modality, and Timing

RRT remains indicated for complications refractory to medical therapy (e.g., severe hyperkalemia, persistent metabolic acidosis, refractory fluid overload, symptomatic uremia) or failure of conservative measures, and modality selection should be individualized based on hemodynamic stability, catabolic state, and local resources [2,3]. Regarding timing, randomized trials in critically ill patients, including sepsis cohorts, support that an accelerated/early strategy has not been shown to improve outcomes compared with a deferred strategy incorporating close monitoring and rescue criteria, suggesting that unnecessary early exposure to RRT may often be avoided when close surveillance is feasible [74,79,80].

### 6.5. Pharmacologic and Targeted Therapies

Targeted interventions remain under investigation. Alkaline phosphatase has been explored in drug-associated AKI as an immunomodulatory strategy through dephosphorylation of endotoxins; however, it is not standard therapy and remains under study [81]. Similarly, approaches targeting mitochondrial dysfunction or regulated cell-death programs (e.g., ferroptosis) remain largely preclinical or in early translational phases, without sufficient evidence for routine implementation [2,3,14].

In drug-associated AKI, management relies on early recognition, dose adjustment, withdrawal of the causative agent, and avoidance of additional nephrotoxins; therapeutic drug monitoring is particularly relevant for nephrotoxic antimicrobials such as vancomycin [49]. In the perioperative setting, preventive bundles focus on hemodynamic stability and avoidance of sustained hypotension, with close postoperative surveillance [6,78,82]. In patients with cirrhosis or malignancy, AKI management depends on phenotype and goals of care, requiring individualized decisions regarding vasoconstrictors/albumin in selected scenarios and initiation of RRT when clinically indicated [64,65,83]. Bedside tools, including point-of-care ultrasound, may facilitate dynamic assessment of volume status and venous congestion, reinforcing physiologic alignment of resuscitation and de-resuscitation decisions [84].

**Figure 2:**
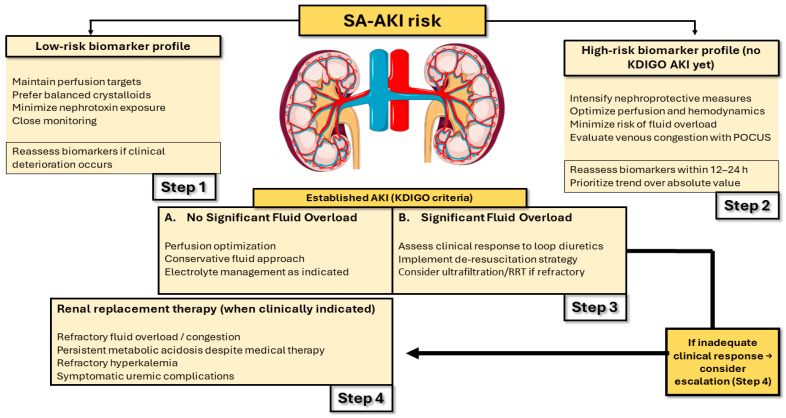
Decision-support framework for SA-AKI integrating biomarker risk profiles (specified in the main text), fluid-status phenotyping, and trajectory-based escalation. High-risk biomarker profiles without KDIGO-defined AKI identify a subclinical risk window prompting intensified nephroprotective measures and early reassessment, whereas established AKI is stratified by fluid overload to guide conservative versus de-resuscitation strategies. Escalation to renal replacement therapy is reserved for clinically indicated, refractory complications. (Adapted from [4,79].)

The overall clinical decision structure is summarized in Figure 2.

## 7. Discussion: Critical Appraisal of the Evidence

This appraisal was developed as a narrative synthesis, structured through an evidence hierarchy using the Oxford Centre for Evidence-Based Medicine Levels of Evidence (OCEBM 2011) [85]. These levels do not replace risk-of-bias assessment; rather, they provide a conceptual backbone to prioritize the inferential strength of findings and to curb unsupported claims. Accordingly, this section integrates (i) Levels of Evidence (LoE); (ii) diagnostic/prognostic performance (AUROC, sensitivity/specificity); (iii) pathophysiological plausibility; and (iv) clinical applicability, explicitly distinguishing evidence generated in sepsis/SA-AKI populations from evidence extrapolated from other AKI etiologies.

In this context, meta-analyses provide pooled estimates of performance, but their interpretation is critically contingent on clinical and methodological heterogeneity (sepsis and AKI definitions, sampling time, specimen matrix, assay platform, and diagnostic thresholds). In SA-AKI, a quantitative synthesis of 49 studies including 4189 patients reported SROC values of 0.931 for urinary KIM-1, 0.907 for urinary NGAL, and 0.861 for urinary IL-18; plasma NGAL showed a sensitivity of 0.77 and specificity of 0.61.

Nevertheless, even at this evidence level, residual sources of variability may constrain external validity: (1) a threshold effect driven by non-standardized cutoffs across studies, (2) spectrum bias due to mixed severity strata (sepsis without shock vs. septic shock; mild vs. moderate-to-severe AKI), and (3) misclassification bias attributable to an imperfect reference standard in patients with rapid volume shifts or changes in endogenous creatinine generation [86].

Consistently, the classic NGAL meta-analysis comprising 19 studies and 2538 patients (19.2% developed AKI) reported an overall AUROC of approximately 0.82, with sensitivity ~0.76 and specificity ~0.85, but with attenuated performance in ICU cohorts (AUROC ~0.73). These findings suggest an interaction between clinical context, pre-test probability, and assay platform [87]. These considerations caution against claims of biomarker “superiority” when they are derived from indirect comparisons. Table 5 summarizes diagnostic performance alongside OCEBM 2011 levels of evidence.

In terms of biomarkers with clinical validation for early risk stratification in critically ill patients, the urinary [TIMP-2]•[IGFBP7] index (NephroCheck) has been assessed in multicenter prospective cohorts with clinically relevant outcomes, specifically moderate-to-severe AKI (KDIGO stages 2–3) within a short time window. In SAPPHIRE (*n* = 728; 14% reached the endpoint), [TIMP-2]•[IGFBP7] identified patients at risk for KDIGO 2–3 within 12 h with an AUROC of ~0.80 [34].

Complementarily, TOPAZ (*n* = 420; 17.4% endpoint) corroborated performance (AUROC ~0.82) and operationalized a high-sensitivity cutoff (0.3), yielding Se 92% and Sp 46% [88]. These operating characteristics imply a sensitivity–specificity trade-off between early detection and false positives in populations with lower pre-test probability. In this context, Vijayan et al. (2016) proposed interpreting [TIMP-2]•[IGFBP7] as a marker of tubular stress and vulnerability (short-term risk of progression) [94].

Two methodological features support the internal validity of these findings: (1) the prospective design, which mitigates selection bias and reduces temporal ambiguity between biomarker measurement and endpoint assessment, and (2) blinded endpoint adjudication in TOPAZ, which decreases misclassification attributable to an imperfect functional reference standard—particularly relevant in critically ill patients experiencing rapid volume shifts, hemodilution, and non-steady-state creatinine kinetics [88].

The practical implication is that [TIMP-2]•[IGFBP7] is optimized for short-term risk stratification rather than etiologic diagnosis; therefore, its moderate specificity at high-sensitivity thresholds supports interpretation within an integrated clinical framework to minimize unnecessary interventions driven by operational false positives [94]. Additionally, although SAPPHIRE enrolled critically ill patients with respiratory and/or cardiovascular dysfunction, the proportion of sepsis was relatively limited (19%; 136/728); accordingly, extrapolation to SA-AKI should be framed cautiously and, when feasible, anchored to evidence generated specifically in septic cohorts [34].

For biomarkers that capture tubular injury (and, in part, associated inflammatory signaling), the evidence base is substantial yet heterogeneous; thus, interpretation must balance biomarker maturity (validation, availability, accumulated experience) against its specificity in septic settings, where a renal signal coexists with a high systemic inflammatory background.

In a systematic review and meta-analysis focused on SA-AKI diagnosis, Xie et al. (2021) [86] reported for uKIM-1 an Se of 0.86 (95% CI: 0.79–0.91) and Sp of 0.84 (95% CI: 0.79–0.88), with an SROC of 0.931; for uNGAL, Se 0.81 (95% CI: 0.77–0.84) and Sp 0.79 (95% CI: 0.76–0.82), with SROC 0.907; for uIL-18, Se 0.80 (95% CI: 0.77–0.82) and Sp 0.70 (95% CI: 0.67–0.72), with SROC 0.861; and for plasma NGAL, Se 0.77 (95% CI: 0.72–0.81) with Sp 0.61 (95% CI: 0.58–0.65). This pattern—particularly the lower specificity of plasma NGAL—is consistent with greater susceptibility of the blood compartment to systemic inflammation, endothelial dysfunction, and other extra-renal sources of variation in sepsis, which may attenuate renal specificity.

Nevertheless, comparisons based on SROC magnitude should be interpreted as pooled statistical summaries rather than definitive clinical superiority: pooled estimates integrate studies with diverse assay platforms, non-uniform sepsis and AKI definitions, and heterogeneous sampling windows; moreover, direct comparative designs evaluating multiple biomarkers within the same patients under equivalent conditions remain relatively scarce. Accordingly, in SA-AKI, biomarker selection should be anchored to the clinical question (early detection, risk stratification, or trajectory), as well as local analytic feasibility and the scenario’s pre-test probability (e.g., sepsis without shock vs. septic shock) [86].

Evidence derived from other AKI settings strengthens the temporal plausibility of these biomarkers; however, given substantial differences in the underlying pathophysiology and inflammatory background, it does not obviate the need for sepsis-specific validation. In TRIBE-AKI, a multicenter prospective cardiac surgery cohort (*n* = 1219), uIL-18 and NGAL (urinary and plasma) reached peak values within the first 6 postoperative hours and provided an incremental gain in discrimination when added to a clinical model, increasing the AUROC from 0.69 to 0.76 for uIL-18, with plasma NGAL AUROC ~0.75 [92].

In addition, a meta-analysis of uIL-18 (11 studies; 2796 patients) estimated an AUROC of ~0.77, with Se 0.51 and Sp 0.79, and described better performance when sampling occurred early (<12 h) [91]. The critical appraisal is twofold: first, sampling time is a structural determinant of apparent performance—early-response biomarkers tend to lose discrimination when quantified late; second, extrapolating kinetics and specificity across injury phenotypes is limited, because perioperative ischemia–reperfusion and sepsis differ in dominant mechanisms, which may shift both temporal dynamics and renal specificity. Accordingly, these data are best interpreted as pathophysiological and design support (i.e., guiding sampling windows), rather than as primary evidence of performance in SA-AKI.

Functional biomarkers may add a complementary dimension to stress or tubular injury markers. In an ICU cohort (*n* = 444; 18% with sepsis; 45% developed AKI; 30-day mortality, 14%), urinary cystatin C showed an AUROC of 0.70 for AKI and 0.80 for sepsis; for AKI, the optimal cutoff was 0.12 mg/L, with Se 0.67 and Sp 0.64, suggesting overall moderate performance [95].

From a methodological standpoint, this study exemplifies a common structural bias in biomarker research: when the endpoint is defined by a creatinine-based functional definition, any biomarker with an earlier rise may appear “falsely positive” due to outcome misclassification. To mitigate this issue, the authors incorporated adjusted multivariable models and reported an AUROC of approximately 0.84 for logistic models predicting AKI and sepsis, which is consistent with an incremental improvement in the model’s discriminatory capacity; however, these findings do not, in themselves, establish actionable clinical utility or demonstrate benefit in patient-centered outcomes, as biomarker-guided management strategies were not evaluated [95].

For penKid, the most relevant evidence in sepsis can be viewed along two complementary lines: physiological validation and prognostic validation. In a pilot septic shock study using measured glomerular filtration based on iohexol clearance as the reference standard (*n* = 23), penKid correlated closely with measured GFR (R^2^ = 0.90), with performance at least comparable—and trending higher—than creatinine-based estimates, supporting its role as a functional estimator under non-steady-state conditions typical of critical illness [89]. Nevertheless, the small sample size and pilot design limit precision, hinder robust assessment of heterogeneity, and thereby constrain external validity.

In 2020, Dépret et al. conducted a prognostic evaluation in a cohort of critically ill patients (*n* = 2004) and an independent septic shock subgroup (*n* = 583), operationalizing the construct of “subclinical AKI”. This phenotype was not uncommon (6.1% and 6.7%, respectively) and was associated with higher 28-day mortality; in the septic shock subgroup, a substantial risk increase was reported (adjusted HR ~2.5) [26].

While the conceptual implication is important, inference warrants methodological caution: the phenotype depends critically on the biomarker cutoff (a potential threshold effect), and the absence of an independent standard for injury/function may introduce outcome misclassification and residual confounding even after multivariable adjustment.

Along a different axis focused on recovery, a post hoc analysis of the ELAIN trial (*n* = 210) suggested that low penKid levels at initiation of renal replacement therapy (RRT) were associated with a higher probability of successful discontinuation of RRT (sHR ~1.8), estimated using a competing-risks framework (Fine–Gray). Despite biological plausibility, the post hoc nature and potential indication bias (who initiates RRT, when, and with what intensity) support classifying this signal as exploratory—useful for hypothesis generation and prospective study design, but insufficient to guide decisions about RRT discontinuation in isolation [90].

A further step in clinical maturity is the shift from early AKI detection toward predicting trajectory (persistence/non-recovery), a domain with greater potential for action because it aligns with ICU support decisions and resource allocation. In RUBY (*n* = 331 in the primary analysis), urinary CCL14 measured after the onset of moderate-to-severe AKI discriminated persistence or progression to severe AKI at 72 h, with an AUROC of 0.83 and performance exceeding that of several comparator biomarkers evaluated within the same analytic framework.

This design may reduce some of the confounding inherent in cross-study comparisons by evaluating biomarkers within a single cohort using harmonized outcome definitions; however, its key implication for applicability is that it is, by construction, a post-AKI biomarker: its informative value emerges once patients have already reached at least KDIGO stage ≥2, thereby informing secondary management decisions (e.g., anticipatory planning for RRT, monitoring intensity, and fluid balance targets) rather than primary prevention interventions [40].

At the exploratory end of the spectrum, miRNAs illustrate a classic gap between molecular plausibility and clinical extrapolability. In a 2024 meta-analysis, Brown et al. integrated a broad repertoire of miRNAs and observed that, although predictive signals were reported, no single miRNA showed consistent replication across more than one study—findings compatible with between-cohort heterogeneity, multiple testing, and the absence of standardized pre-analytic/analytic protocols [96].

Similarly, a 2025 validation study in SA-AKI by Van der Aart et al. reported modest performance for miR-21-5p (AUROC ~0.68 in an emergency department cohort and 0.74 in an ICU cohort), with limited prognostic utility in real-world clinical settings [93]. In this context, and within the OCEBM 2011 framework, these biomarkers should be classified as exploratory: incorporation into clinical recommendations would remain premature without multicenter validation, prespecified protocols (including sampling windows, normalization procedures, and thresholds), and explicit evaluation of incremental performance beyond standard clinical models [93,96]. Table 6 organizes biomarkers according to evidentiary robustness and the extent of clinical validation.

**Table 6:** Clinical readiness and validation context of biomarkers in SA-AKI.

Clinical Readiness Level	Biomarker	Specimen	Supporting Evidence	Population/Setting
Robust clinical validation for early risk stratification	[TIMP-2]•[IGFBP7] (NephroCheck)	Urine	Kashani et al., 2013 [34]; Bihorac et al., 2014 [88]; Vijayan et al., 2016 [94]; Ostermann et al., 2020 [97]	Mixed ICU; subgroup/scenarios with sepsis (not exclusively SA-AKI across the full evidence base)
Diagnostic evidence in SA-AKI	KIM-1	Urine	Xie et al., 2021 [86]	Direct SA-AKI evidence (sepsis-specific synthesis)
Diagnostic evidence in SA-AKI	NGAL	Urine/plasma	Haase et al., 2009 [87]; Xie et al., 2021 [86]; Parikh et al., 2011 [92]	Direct SA-AKI evidence [86] + extrapolation from general/surgical AKI [87,92]
Diagnostic evidence in SA-AKI	IL-18	Urine	Xie et al., 2021 [86]; Lin et al., 2015 [91]; Parikh et al., 2011 [92]	Direct SA-AKI evidence [86] + extrapolation from general/surgical AKI [91,92]
Functional validation in critically ill patients	UCysC	Urine	Nejat et al., 2010 [95]	ICU cohort with a septic fraction (not SA-AKI-specific)
Trajectory/persistence validation	CCL14	Urine	Hoste et al., 2020 [40]	Moderate-to-severe AKI in ICU (not exclusively sepsis)
Mixed evidence: physiological + prognostic	penKid	Plasma	Beunders et al., 2020 [89]; Dépret et al., 2020 [26]; von Groote et al., 2022 [90]	Sepsis/septic shock and critically ill patients; RRT in AKI
Exploratory/emerging	miRNAs	Plasma (study-dependent)	Brown et al., 2024 [96]; Van der Aart et al., 2025 [93]	SA-AKI (validation) + general AKI (synthesis)

Note: Biomarkers are organized by clinical readiness; this ordering does not imply superiority. Specimen type, supporting evidence, and population context are provided.

Overall, the prioritization of evidence for SA-AKI can be synthesized into five domains, ordered by clinical readiness and proximity to implementation: first, [TIMP-2]•[IGFBP7] as an early risk-stratification tool in critically ill patients, supported by multicenter prospective validations and implementation-focused research [34,88,94]; second, tubular injury biomarkers with quantitative evidence specifically in sepsis—uKIM-1, uNGAL, and uIL-18—which may support early diagnosis and risk stratification but remain context-dependent [86]; third, functional biomarkers (uCysC, penKid) that provide a complementary readout of kidney function and subclinical/prognostic phenotypes [26,89,95]; fourth, trajectory/persistence markers, with CCL14 as a candidate supported by multicenter data [40]; and finally, exploratory biomarkers such as miRNAs, where heterogeneity and lack of standardization constrain clinical translatability [93,96]. In parallel, the ADQI 23 consensus emphasized that moving toward implementation requires, beyond diagnostic performance, evidence of clinical utility to guide decisions and operational integration into care pathways [97].

### 7.1. Temporal Implications and Proposed Sampling Time Points

Based on this critical appraisal, selection of sampling time points can be anchored to a pathophysiologically coherent index time point and linked to specific clinical decisions. Table 7 summarizes the indicative time windows for biomarker rise and peak that underpin the subsequent sampling algorithm.

Below, we propose a stepwise sampling algorithm to integrate biomarkers into the assessment of SA-AKI. The aim is to operationalize the “when” and the “why” of each measurement, rather than to replace conventional criteria, and it should not be interpreted as implying prescriptive superiority among biomarkers. The framework is anchored to an index time point (T0) and successive pathophysiological windows (stress, injury, trajectory), so that each biomarker is interpreted according to its predominant biological domain and the corresponding clinical decision point (intensified surveillance and kidney-protective measures, early diagnostic support, or stratification of persistence/non-recovery).

### 7.2. Prototype Algorithm for Longitudinal Sampling in SA-AKI

Purpose and scope. This algorithm is intended for use in research cohorts and/or ICU protocols to operationalize sampling windows aligned with pathophysiological domains. It is not intended to replace KDIGO criteria or to serve as a standalone trigger for therapeutic decisions.

Step 1. Define the index time point (T0).

T0 is defined as the time of clinical recognition of sepsis (e.g., Sepsis-3) or, when onset is uncertain, ICU admission.

Step 2. Time-stratified sampling windows.

*Early stress window (T6–12 h from T0)*: [TIMP-2]•[IGFBP7]

Objective: Identify patients in a tubular stress phase at short-term risk of progressing to moderate-to-severe AKI [34,88,94].

*Injury/function confirmation window (T12–24 h)*: uNGAL + penKid

Objective: Capture a tubular injury signal and, in parallel, estimate the functional dimension under non-steady-state conditions typical of critical illness [26,86,89].

*Trajectory window (T48–72 h)*: CCL14 ± KIM-1

Objective: Stratify persistence/progression once moderate-to-severe AKI is established, and optionally complement with a tubular injury marker if the protocol aims for serial phenotypic characterization [40,86].

Step 3. Decision tree (operational interpretation).

*If T6–12 h and [TIMP-2]•[IGFBP7] ≥ 0.3*: Consider implementing kidney-protective measures and intensifying surveillance (fluid balance optimization, nephrotoxin review, close monitoring, and hemodynamic reassessment), recognizing that biomarker elevation alone does not establish injury nor mandate escalation independent of clinical context [88,94].

*If <0.3*: Continue standard monitoring and reassess according to clinical evolution if instability persists.

*If T12–24 h and penKid ≥ 80 pmol/L in the absence of conventional functional criteria*: Consider escalation of monitoring and risk assessment, while acknowledging that threshold-dependent classification may vary across settings and requires protocol standardization [26]. In parallel, a positive/elevated uNGAL supports the presence of tubular injury in sepsis, with interpretation contingent on specimen matrix, inflammatory phenotype, sampling time, and assay characteristics [86].

*If T48–72 h and CCL14 are elevated (protocol-defined threshold)*: Suggest increased risk of persistence/progression and inform secondary management decisions (monitoring intensity, fluid balance targets, and anticipatory planning for RRT if the clinical trajectory suggests it), while avoiding use of the biomarker as the sole criterion to initiate RRT [40].

*Note*: This algorithm emphasizes that biomarker-based evaluation should be longitudinal and anchored to pathophysiological windows. Aligning sampling with the predominant biological domain (stress, injury, trajectory) may reduce misinterpretation associated with late or isolated measurements and facilitates integration with clinical decision making and KDIGO criteria; implementation should ultimately be supported by evidence of clinical utility within care pathways.

In summary, the available evidence supports a stratified, context-dependent approach to biomarkers in SA-AKI: prioritizing those with greater clinical maturity and validation for the specific objective; explicitly separating sepsis-derived evidence from extrapolated data; and orienting implementation toward demonstrable clinical utility through longitudinal sampling integrated into care pathways, rather than toward unsupported claims of “superiority.”

## 8. Future Research Directions

The available evidence indicates that acute kidney injury (AKI) is a biologically heterogeneous syndrome and is likely to benefit from integrative approaches linking molecular characterization, biomarker refinement, and phenotype-oriented clinical investigation.

At the molecular level, single-cell transcriptomic and epigenomic studies have identified that AKI encompasses distinct cellular states, including adaptive stress responses, failed repair programs, and maladaptive reprogramming [7,11,24]. Whether reproducible molecular signatures can be translated into clinically scalable tools remains to be determined. Such translation will require standardized sampling strategies, assay harmonization, reproducibility across platforms, and rigorous external validation in independent cohorts.

In parallel, multimodal biomarker strategies warrant prospective evaluation in well-phenotyped populations. Rather than assessing isolated markers, future studies should examine integrated panels spanning structural, functional, inflammatory, and molecular domains, using prespecified protocols and quantifying incremental discrimination beyond established clinical models [3,4]. Particular emphasis should be placed on phenotype-specific signatures (e.g., sepsis, ischemia–reperfusion, nephrotoxicity), markers of maladaptive repair, and predictors of the AKI→CKD transition.

Therapeutic development remains closely tied to mechanistic stratification. Preclinical and early translational data implicate mitochondrial dysfunction, microvascular injury, glycocalyx degradation, regulated necrosis pathways (e.g., RIPK1/RIPK3 signaling), ferroptosis, and innate immune activation as contributors to persistent injury [6,7,45,59]. Determining whether modulation of these pathways alters clinically meaningful outcomes will require adequately powered trials incorporating biomarker-defined phenotypes and mechanistically aligned interventions.

Artificial intelligence and predictive modeling represent complementary tools for risk estimation and longitudinal trajectory prediction. However, clinical implementation requires multicenter validation, assessment of calibration and transportability, mitigation of overfitting and dataset shift, transparency in model architecture, integration into structured clinical workflows, and evaluation of clinical impact [86].

Finally, future clinical trials should move beyond treating AKI as a uniform entity and instead incorporate biologically informed enrichment strategies. Targeting inflammatory, hemodynamic, toxic, or maladaptive-repair phenotypes may enhance therapeutic signal detection and improve interpretability. Such designs should align biomarker-defined subgroups with interventions directed at underlying mechanisms rather than relying exclusively on late functional surrogates.

Collectively, sustained progress in AKI research is likely to depend on alignment of molecular insights, robust biomarker validation, phenotype-guided trial design, and demonstration of clinical utility within rigorously evaluated implementation frameworks.

## 9. Conclusions

Sepsis-associated acute kidney injury is a biologically and clinically heterogeneous entity in which hemodynamic alterations, systemic inflammation, endothelial dysfunction, microvascular injury, and maladaptive repair interact across dynamic temporal windows. The accumulated evidence indicates that SA-AKI may not be adequately characterized by functional criteria alone and benefits from integration of molecular, structural, and trajectory-based information.

Across currently available tools, biomarker evidence and applicability remain uneven. Urinary [TIMP-2]•[IGFBP7] has among the most consistent prospective validation for short-term risk stratification in critically ill populations; tubular injury and functional markers (e.g., uNGAL/uKIM-1/uIL-18; penKid/uCysC) provide complementary information that is context-dependent in sepsis, and trajectory biomarkers such as CCL14 add prognostic value once moderate-to-severe AKI is established. In contrast, exploratory molecular signatures (including circulating miRNAs) require further standardization and external validation before clinical translation.

Accordingly, biomarker use in SA-AKI is best approached as stratified and longitudinal—anchored to pathophysiological windows and integrated with KDIGO criteria—while implementation should be guided by demonstrated clinical utility in prospective studies, including incremental value beyond established models and impact on patient-centered outcomes.

Sustained progress in SA-AKI management will likely depend on aligning mechanistic phenotyping, rigorously validated biomarker strategies, and phenotype-guided clinical trials within structured implementation frameworks, while avoiding unsupported claims of superiority and remaining aligned with evidence-based practice.

## Figures and Tables

**Table 2:** Classification according to biomarker type in AKI.

Biomarker Category	Biomarkers
Tubular injury markers	NGAL, KIM-1, IL-18, L-FABP
Cellular stress markers	TIMP-2, IGFBP7
Renal function markers	Cystatin C, penKid
Inflammatory markers	CCL14

Adapted from [22].

**Table 3:** Early biomarkers in AKI.

Biomarker	Clinical Use
CysC	Prediction; Diagnosis
penKid	Diagnosis; Severity
[TIMP-2]•[IGFBP7]	Prediction
NGAL	Prediction; Diagnosis; Severity
KIM-1	Diagnosis
Urinary IL-18	Prediction; Diagnosis
Urinary L-FABP	Prediction
Urinary sTREM-1	Prediction; Diagnosis; Severity

Adapted from [4,6,8,14,16,23,24,25,26]. Abbreviations: CysC = cystatin C; penKid = proenkephalin A 119–159; TIMP-2 = tissue inhibitor of metalloproteinases 2; IGFBP7 = insulin-like growth factor–binding protein 7; [TIMP-2]•[IGFBP7] = urinary product of both stress biomarkers; NGAL = neutrophil gelatinase-associated lipocalin; KIM-1 = kidney injury molecule 1; IL-18 = interleukin 18; L-FABP = liver-type fatty acid–binding protein; sTREM-1 = soluble triggering receptor expressed on myeloid cells 1.

**Table 4:** Conceptual framework integrating trajectories, phenotypes, endotypes, and biomarker domains in sepsis-associated AKI.

Framework	Categories	Operational Definition	Practical Use
AKI trajectory phenotypes	No AKI; rapidly reversed AKI; persistent AKI with renal recovery; persistent AKI without renal recovery	Clinical trajectories defined using ADQI/KDIGO criteria (reversibility, persistence, and recovery).	Guides monitoring intensity and recovery-centered endpoints; identifies highest-risk trajectory (persistent AKI without recovery).
Temporal phenotype of SA-AKI	Early SA-AKI Late SA-AKI	Early: AKI ≤ 48 h from sepsis diagnosis; late: AKI 48 h–day 7.	Frames SA-AKI as dynamic; supports contextualization of timing, “trajectory,” and potential therapeutic windows.
Endotypes/subphenotypes in sepsis with AKI	Endotype 1 (low endothelial dysfunction/inflammation); Endotype 2 (high endothelial dysfunction/inflammation); AKI-SP1/AKI-SP2	Latent class-derived classes; higher endothelial/inflammatory signals in high-risk groups.	Trial enrichment/stratification; helps interpret heterogeneity of treatment effect (e.g., post hoc signal with vasopressin in SP1); not framed as routine bedside endotyping.
Biomarker-based prognostic indicators	penKid > 80 pmol/L at admission without AKI criteria; [TIMP-2]•[IGFBP7] > 0.3 post resuscitation	“Hidden high-risk” states detected; [TIMP-2]•[IGFBP7] assessed before/after early resuscitation.	Identifies occult risk of progression/severe AKI/dialysis/death; supports reassessment after resuscitation and trajectory-based monitoring.
Sepsis phenotypes (Seymour framework)	α, β, γ, δ phenotypes	Data-derived sepsis phenotypes; β/γ shows greater renal dysfunction; δ may concentrate worse prognosis/resource use.	Supports the concept that SA-AKI risk and expression differ by sepsis phenotype.

Adapted from [72,75,76]. Abbreviations: AKI, acute kidney injury; SA-AKI, sepsis-associated AKI; ADQI, Acute Disease Quality Initiative; KDIGO, Kidney Disease: Improving Global Outcomes.

**Table 5:** Performance and level of evidence (OCEBM 2011) for selected biomarkers relevant to SA-AKI.

Biomarker	Specimen	Endpoint	Primary Metric	Se/Sp (If Applicable)	Study Design	LoE (OCEBM 2011)	Reference
uKIM-1	Urine	SA-AKI diagnosis	SROC 0.931	Se 0.86; Sp 0.84	Systematic review + meta-analysis	**1a**	Xie et al., 2021 [86]
uNGAL	Urine	SA-AKI diagnosis	SROC 0.907	Se 0.81; Sp 0.79	Systematic review + meta-analysis	**1a**	Xie et al., 2021 [86]
uIL-18	Urine	SA-AKI diagnosis	SROC 0.861	Se 0.80; Sp 0.70	Systematic review + meta-analysis	**1a**	Xie et al., 2021 [86]
NGAL (plasma)	Plasma	SA-AKI diagnosis	(Quantitative synthesis)	Se 0.77; Sp 0.61	Systematic review + meta-analysis	**1a**	Xie et al., 2021 [86]
[TIMP-2]•[IGFBP7]	Urine	AKI risk	AUROC ~0.80	—	Multicenter prospective cohort	**1b**	Kashani et al., 2013 [34]
[TIMP-2]•[IGFBP7]	Urine	AKI risk	AUROC ~0.82	Se 0.92; Sp 0.46	Multicenter prospective cohort	**1b**	Bihorac et al., 2014 [88]
penKid	Plasma	GFR validation	R^2^ ~0.90	—	Physiological study	**2b**	Beunders et al., 2020 [89]
penKid	Plasma	28-day mortality	Adjusted HR ~2.5	—	Observational cohort	**2b**	Dépret et al., 2020 [26]
penKid	Plasma	RRT liberation	sHR ~1.83	—	Post hoc trial analysis	**3b**	von Groote et al., 2022 [90]
CCL14	Urine	Persistent AKI	AUROC 0.83	—	Multicenter observational cohort	**1b**	Hoste et al., 2020 [40]
NGAL	Urine/plasma	AKI diagnosis	AUROC 0.815	Se 76.4%; Sp 85.1%	Systematic review + meta-analysis	**1a**	Haase et al., 2009 [87]
uIL-18	Urine	AKI diagnosis	AUROC ~0.77	Se 0.51; Sp 0.79	Systematic review + meta-analysis	**1a**	Lin et al., 2015 [91]
IL-18 (±NGAL)	Urine/plasma	Postoperative AKI	Incremental AUROC	—	Multicenter observational cohort	**1b**	Parikh et al., 2011 [92]
miR-21-5p	Plasma	SA-AKI validation	AUROC ~0.68–0.74	—	Cohort/validation	**2b**	Van der Aart et al., 2025 [93]

Abbreviations: RRT, renal replacement therapy; GFR, glomerular filtration rate; AUROC, area under the receiver operating characteristic curve; ROC, receiver operating characteristic; SROC, summary receiver operating characteristic; Se, sensitivity; Sp, specificity; HR, hazard ratio; sHR, subdistribution hazard ratio; OCEBM, Oxford Centre for Evidence-Based Medicine; LoE, level of evidence. Note: LoE (OCEBM 2011) was assigned according to the primary endpoint. Performance metrics (AUROC/SROC, R^2^, HR/sHR) are not directly comparable across domains.

**Table 7:** Temporal characteristics of key biomarkers in SA-AKI: onset of elevation and peak window to guide sampling.

Biomarker	Specimen	Onset of Elevation	Peak Window	References
[TIMP-2]•[IGFBP7]	Urine	6–12 h from T0	≈12 h (short window)	[34,88,94]
NGAL	Urine	12–24 h (T0-dependent)	≈6–24 h (more defined in insult-delimited settings; T0/phenotype/platform-dependent)	[86,87,92]
KIM-1	Urine	Hours–days (sampling-time dependent)	≈24–48 h	[86]
IL-18	Urine	<12 h	Hours–24 h (variable; sampling-time-dependent)	[86,91,92]
uCysC	Urine	T0 (admission) and/or first hours (protocol-dependent)	Not standardized	[95]
penKid	Plasma	T0–first hours	Variable	[26,89,90]
CCL14	Urine	Post-AKI (KDIGO ≥ 2)	≈72 h	[40]

Note: T0 denotes the index time point (e.g., sepsis recognition or ICU admission). Time windows are indicative and may vary by clinical phenotype, specimen matrix, and assay platform.

## Data Availability

No new datasets were generated or analyzed in this study. This article is a narrative review based on previously published literature, and all relevant data are contained within the article and its cited references.
